# Properties of Macadam Stabilized with Cement and Asphalt Emulsion

**DOI:** 10.3390/ma16237256

**Published:** 2023-11-21

**Authors:** Jian Sun, Yong Huang, Yulin He, Bieliatynskyi Andrii, Wen Liu

**Affiliations:** 1 State Key Laboratory of Chemistry and Utilization of Carbon Based Energy Resources, College of Chemistry, Xinjiang University, Urumqi 830017, China; sunjian2021@gmail.com (J.S.); liuwen20000324@163.com (W.L.); 2 Department of Automobile Road Construction and Maintenance, Kharkiv National Automobile and Highway University, Yaroslava Mudrovo Street, 25, 61002 Kharkiv, Ukraine; 3 School of Transportation Science and Engineering, Harbin Institute of Technology, Xidazhi Street 92, Harbin 150090, China; yulinhe@hit.edu.cn; 4 School of Civil Engineering, North Minzu University, 204 Wenchang Road, Yinchuan 750021, China; beljatynskij@ukr.net

**Keywords:** cement-stabilized macadam, asphalt emulsion, unconfined compressive strength, frost resistance, flexural strength, elastic modulus

## Abstract

The cracking of cement-stabilized macadam (CSM) reflects to the asphalt layer, which is one of the reasons for the failure of pavement performance and structure. Adding asphalt emulsion to CSM can effectively prevent the formation of cracks. The primary purpose of this article is to reveal the effect of asphalt emulsions on the performance of CSM by adding different contents of asphalt emulsion. For this purpose, tests of unconfined compressive strength (UCS), flexural tensile strength (FTS), elastic modulus, and frost resistance were performed on CSM with gradations of CSM-5 and CSM-10 (the maximum particle sizes of the macadam in the gradation composition are 5 mm and 10 mm), respectively. The test results showed that the UCS of CSM decreased with the increment of asphalt emulsion content. The FTS and elastic modulus of CSM increased with the content of asphalt emulsion. Based on the FTS test results, the frost resistance coefficient K_m1_, defined according to the CSM splitting strength prior to and subsequent to freeze–thaw, was used to evaluate the frost resistance. The test results showed that the frost resistance of CSM improved with the increase in asphalt emulsion content for the same cement content. In conclusion, adding asphalt emulsion to CSM has positive effects on the FTS, elastic modulus, and frost resistance. Therefore, for the purpose of maintaining the UCS value of CSM, the content of cement should be considered at the same time as the controlling of the content of asphalt emulsion.

## 1. Introduction

A semi-rigid base has the advantages of high strength, good integrity, and high load capacity [1]. Therefore, it has been widely used in road base engineering [2,3]. Cement-stabilized macadam (CSM) is a classical semi-rigid base material. CSM generally has high strength and good durability after curing due to the setting action of cement [4,5]. The CSM layer has a better traffic load distribution compared to the granular layer, which means that the asphalt surface layer bears less tensile strain or stress [6,7]. Hence, a CSM base can enhance the asphalt pavement load-bearing capacity without increasing the overall thickness of the pavement layers [8,9].

Many studies have shown that the higher cement content can increase shrinkage cracking in CSM [10,11,12]. The cracking of the CSM layer may be due to dry shrinkage and temperature shrinkage in the cement mortar [13]. The influence of vehicle load and ambient temperature have been shown to cause a significant amount of reflective cracks in the asphalt surface layer, gradually [14,15]. Reflection cracks accelerate the deterioration of pavement structural properties [16]. Thus, the inherent defects in CSM should be minimized in the mixture design.

At present, there are various measures to prevent and control cracks in a CSM base, such as optimizing the gradation of the aggregate [17], controlling the cement content [18], adding fly ash [19], improving the construction quality [20], enhancing the early-age curing condition [21], adding fibers [1], and laying geotextiles [22,23]. Although these measures have improved the crack resistance of CSM bases, the problem of reflective cracking in pavements has still not been solved using CSM itself.

Cement-and-asphalt-emulsion-composite-stabilized-macadam is increasingly used as a composite material in engineering practice [24]. In cement-and-asphalt-emulsion-composite-stabilized-macadam, cement hydration products and asphalt are interwoven and wrapped with mineral materials, forming a three-dimensional network where they cross each other, forming a composite material with cement as the continuous phase and asphalt as the dispersed phase after mixing [25]. Cement-and-asphalt-emulsion-composite-stabilized-macadam can inhibit the generation of reflection cracks and extend the service life of the road. Du studied the mechanical properties and dry shrinkage properties of cement and asphalt emulsion composite stabilized macadam, and the results showed that the addition of asphalt emulsion reduced the dry shrinkage coefficient of CSM and improved the indirect tensile strength [26]. Zhang studied the mechanical performances and compressive resilience modulus of cement-and-asphalt-emulsion-composite-stabilized-macadam, and the results indicated that the compressive strength was reduced by 15–25%, the splitting strength by 21–23% and the flexural tensile strength by 0–10% when the asphalt emulsion content was 2% [27]. Oruc et al. investigated the shrinkage properties of cement-and-asphalt-emulsion-composite-stabilized-macadam and showed that the addition of 2.5%–3% asphalt emulsion reduced the coefficient of temperature shrinkage by 10–18% [28]. Fu analyzed the flexural tensile strength of the CSM, and the results showed that the inclusion of asphalt emulsion could effectively improve the FTS [29]. Jia Kecong [30] studied the influence of asphalt emulsion on the mechanical properties of CSM. With the increase in asphalt emulsion content, the flexibility of CSM increased, and the compressive strength decreased. However, the splitting strength of CSM with asphalt emulsion was increased by 23.5%.

In general, there are relatively few studies on the mechanical properties and frost resistance of cement-and-asphalt-emulsion-composite-stabilized-macadam in the world, domestically or internationally, and the comprehensive evaluation of the mechanical properties and frost resistance of cement and asphalt emulsion composite stabilized aggregates is not sufficient. In this paper, the effects of different asphalt emulsion contents on the unconfined compressive strength (UCS), flexural tensile strength (FTS), elastic modulus, and frost resistance of CSM were systematically studied, and the performance of cement-and-asphalt-emulsion-composite-stabilized-macadam was comprehensively evaluated. This paper has a certain reference value with regard to the application of asphalt emulsion in CSM.

## 2. Materials

### 2.1. Cement

CSM is usually composed of cement, coarse aggregate and fine aggregate. The cement used in this study is M400 Portland cement by Balakrisky Cement Plant, which meets the requirements [31]; its properties are shown in Table 1.

### 2.2. Aggregate

The coarse and fine aggregates are mainly granite aggregates with particle sizes of 0–5 mm and 5–10 mm, which are most commonly used in road construction engineering in Ukraine. The physical properties of the granite were tested according to the requirements of DSTU B B.2.7-46 [32], and the results are shown in Table 2.

### 2.3. Asphalt Emulsion

Due to the low FTS and computational properties (elastic modulus) of CSM, the performance of CSM was improved using cement and cationic asphalt emulsion composite with stabilized macadam. The asphalt emulsion consists of water, asphalt droplets, and emulsifiers; its physical properties are shown in Table 3.

## 3. Mixture Design and Test Methods

### 3.1. Mixture Design 

**Figure 1:**
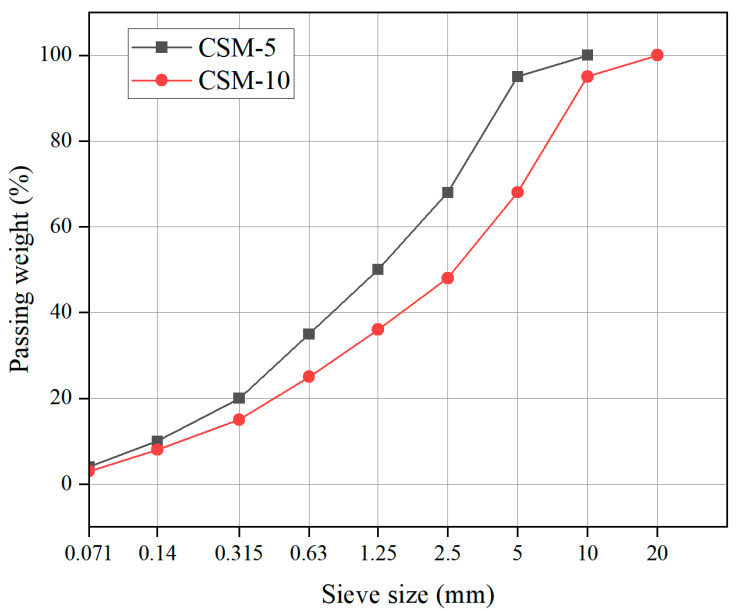
Gradation curves of the mixtures.

In order to study the effect of the asphalt emulsion content on CSM, the cement content was 2%, 4%, 6%, 8%, and 10% of the total aggregate weight, respectively. The selection content of asphalt emulsion is 0%, 2%, 4%, 6%, 8%, and 10% by weight of the total aggregates. In this study, two gradation compositions, CSM-5 and CSM-10 (shown in Figure 1), were used to study the effect of the asphalt emulsion content on the performance of CSM.

### 3.2. Unconfined Compressive Strength

**Figure 2:**
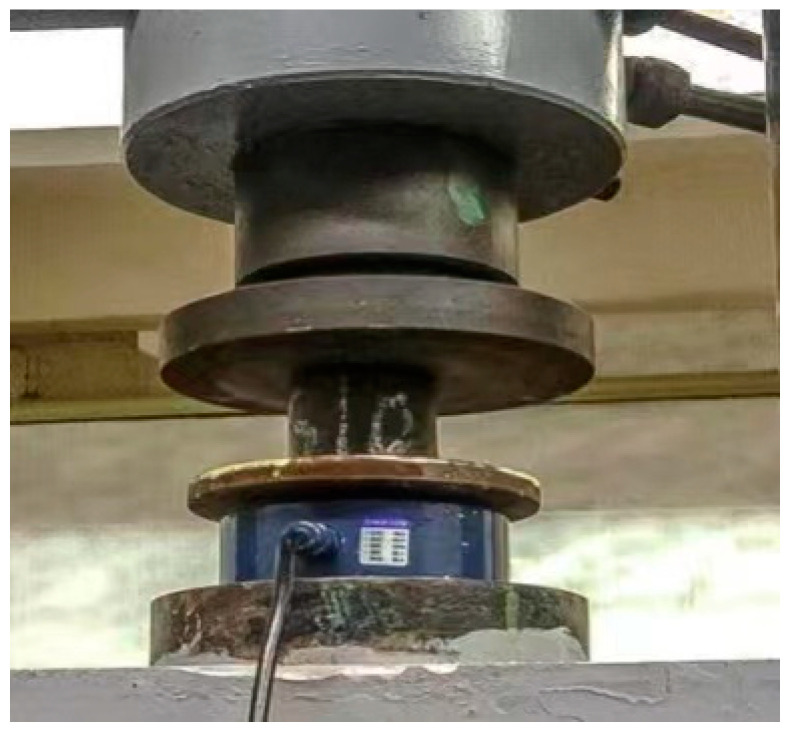
Unconfined compressive strength test.

The specimens were prepared via the static pressure method. The specimens were cylindrical, with a diameter of 100 mm and height of 100 mm. The mold was placed on the compactor, and the mixture was compacted for 3 min at a compaction load of 160 kN. After the specimens were formed, the specimens were placed at a temperature of 20 ± 2 °C and a humidity of 95% for 28 d. On the last day of curing, the specimens were soaked in water for 48 h for UCS tests. The specimens were tested using the P-20 press and CAS MMS-5T force transducer to improve the accuracy of the measurements. The specimen was placed on the press, and pressure was applied at a loading rate of 3 mm/min, until the specimen was broken and the maximum load was recorded, as shown in Figure 2. The results were the average values of the three repetitive specimens from each specific combination.

### 3.3. Flexural Tensile Strength

**Figure 3:**
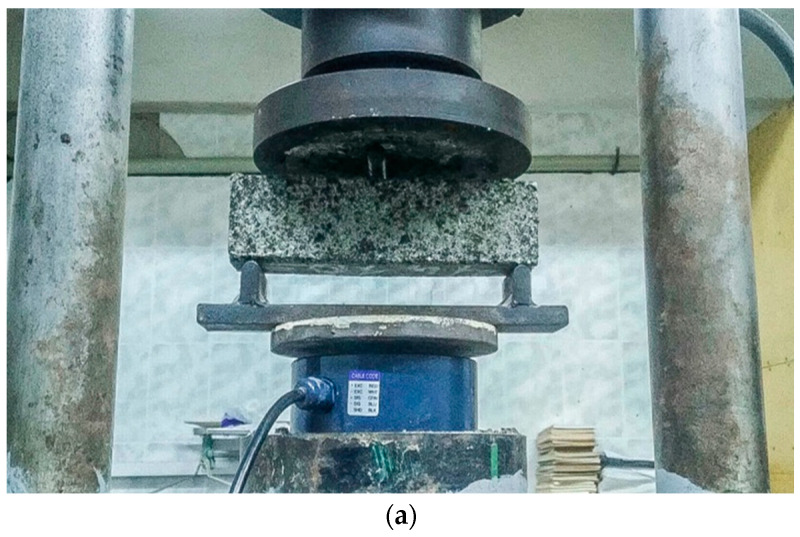

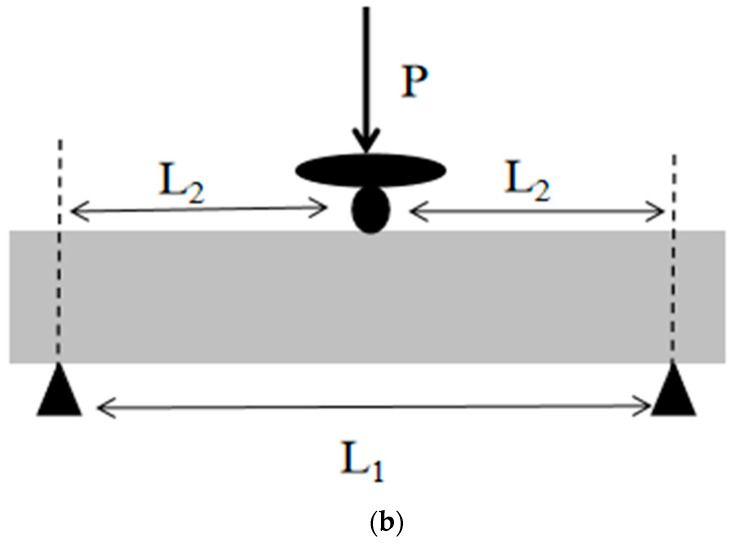
Flexural tensile strength test: (**a**) flexural and tensile strength test; (**b**) flexural and tensile strength test diagram.

The FTS test used 40 × 40 × 160 mm trabecular specimens. The specimens were cured for 28 days at a temperature of 20 ± 2 °C and a humidity of 95%. On the last day of curing, the specimens were soaked in water for 48 h, and then FTS tests were carried out. As shown in Figure 3, both ends of the specimen were fixed on the base, and a stress point was set in the middle position above the specimen, and pressure was applied to it at a rate of 3 mm/min until the specimen fractured. The FTS was calculated as the arithmetic mean of the test results for three specimens; the discrepancy between the results of the individual tests did not exceed 15%.

### 3.4. Elastic Modulus

**Figure 4:**
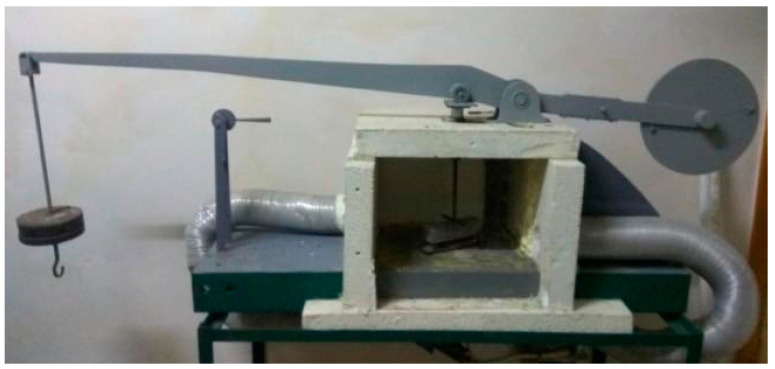
Lever press.

The trabecular specimens cured for 28 d were soaked in water for 24 h to test their elastic modulus. We use a lever press to measure the elastic modulus, as shown in Figure 4. When the elastic modulus test is carried out, the specimens should be loaded in a graded manner. The load level values are set to 3 levels, each with a loading duration of 1 min and then unloading for 30 s. The vertical deflection value of the beam, f_sum_, is measured with an electronic indicator.

The elastic modulus was determined at a load of 0.5–0.7 of the breaking load, according to Formula (1) at *K_t_* = 1.0 and *K_l_* = 1.0:

(1) $$E_{S}=\frac{K_{l}K_{t}Pl^{3}}{48fJ}$$

*K_l_, K_t_*—correction values.

*P*—vertical load, N;

*l*—beam length, 140 mm;

*f*—elastic deflection of the beam, mm;

*J*—moment of inertia of the sample cross section (*J* = bh^3^/12, b, h—the width and height of the beam, respectively), mm^4^.

### 3.5. Frost Resistance

Winters in Ukraine are long and cold, and spring comes late. In the thawing process of winter and early spring, due to the large temperature difference between day and night, after several freeze–thaw cycles, the semi-rigid base course is susceptible to freeze–thaw failure, resulting in melt settling and frost heave. The residual strength ratio after different freeze–thaw cycles was used to evaluate the frost resistance of cement-and-asphalt-emulsion-composite-stabilized-macadam [33,34]. Before the start of the freeze–thaw cycle, the cylindrical specimen of 100 × 100 mm cured for 28 d was soaked with water in a 5% brine solution for 48 h. The specimens were frozen in a low-temperature test chamber at −20 °C for 20 h, and then thawed in salt water at +20 °C for 4 h, which is a freeze–thaw cycle. According to the strength grade of the CSM, different numbers of freeze–thaw cycles were carried out.

## 4. Results and Discussion

### 4.1. Unconfined Compressive Strength

#### 4.1.1. Influence of the Asphalt Emulsion Content

**Figure 5:**
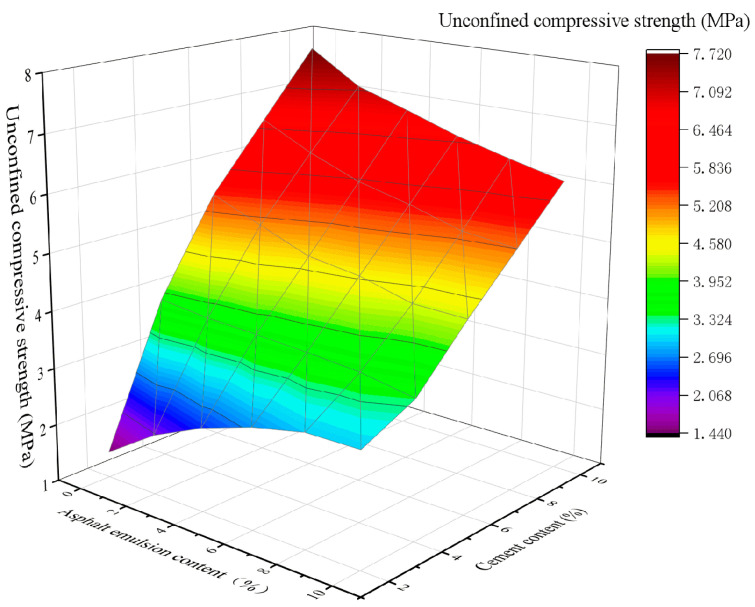
The 3D fitting of the asphalt emulsion content, cement content, and UCS of CSM-5.

**Figure 6:**
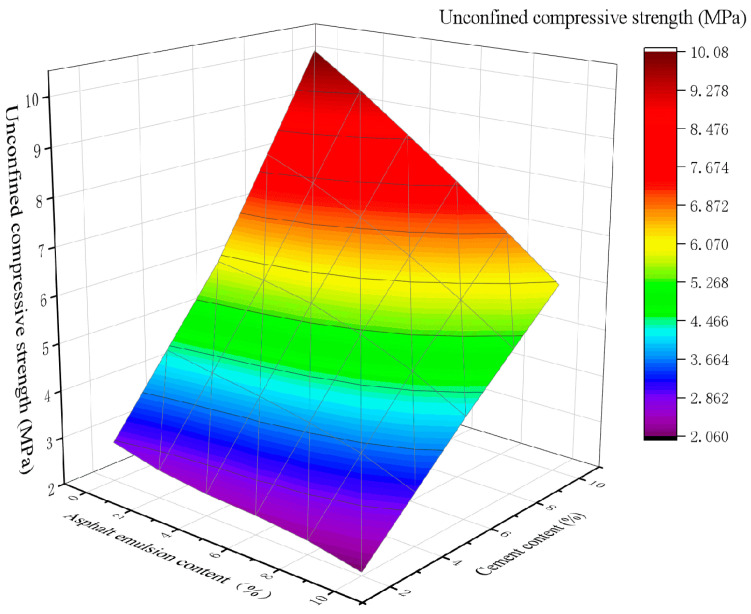
The 3D fitting of the asphalt emulsion content, cement content, and UCS of CSM-10.

To study the effect of the asphalt emulsion content on CSM, 2%, 4%, 6%, 8%, and 10% contents of asphalt emulsion were added to stabilized materials of CSM-5 and CSM-10 with different cement contents, and the test results are shown in Figure 5 and Figure 6.

As can be seen in Figure 5, for the mixture of CSM-5 with 2% cement content, the UCS increased twofold as the asphalt emulsion content increased from 0% to 10%. In contrast, the UCS of CSM-5 mixtures with cement contents of 4%, 6%, 8%, and 10% decreased by 0.14, 1.26, 1.26, and 1.27 times when the asphalt emulsion content was 4%, 6%, 8%, and 10%, respectively. As can be seen from Figure 6, when the cement content was 2%, 4%, 6%, 8% and 10%, the UCS of CSM-10 decreases by 1, 37, 1, 41, 1, 44, 1, 58 and 1.67 times, respectively, as the asphalt emulsion content increases from 0% to 10%. The addition of asphalt emulsion significantly reduced the UCS value of CSM. As the asphalt emulsion content increased, the UCS value gradually decreased. Moreover, the degree of negative impact of asphalt emulsion on UCS was related to the cement content, and the greater the cement content, the greater the decreasing trend of its UCS. The main reason may be that the asphalt film formed by the broken asphalt droplets wraps the cement particles and hinders the hydration of the cement particles. Moreover, as the asphalt emulsion content increased, excessive asphalt emulsion content may form weak interfaces within the CSM, resulting in increased flexibility and weakened UCS in the CSM.

In addition, in the case of the same content of asphalt emulsion, whether in the mixture of CSM-5 or CSM-10, the UCS is proportional to the amount of cement. For all asphalt emulsion contents, the UCS of CSM-5 and CSM-10 mixtures is mainly proportional to the cement content. Therefore, for the purpose of maintaining the UCS value of CSM, the content of cement should be considered at the same time as the controlling of the content of asphalt emulsion.

#### 4.1.2. Influence of Curing Periods

**Figure 7:**
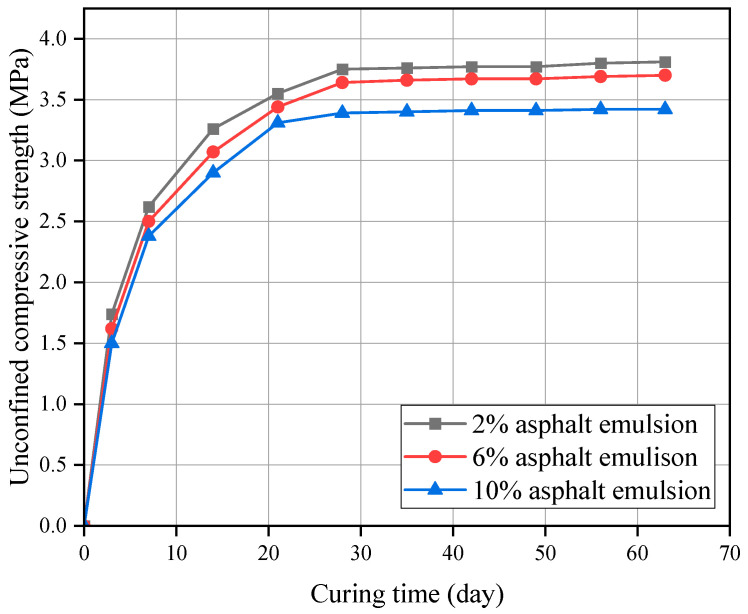
UCS of CSM-5 with 4% cement at different curing periods.

**Figure 8:**
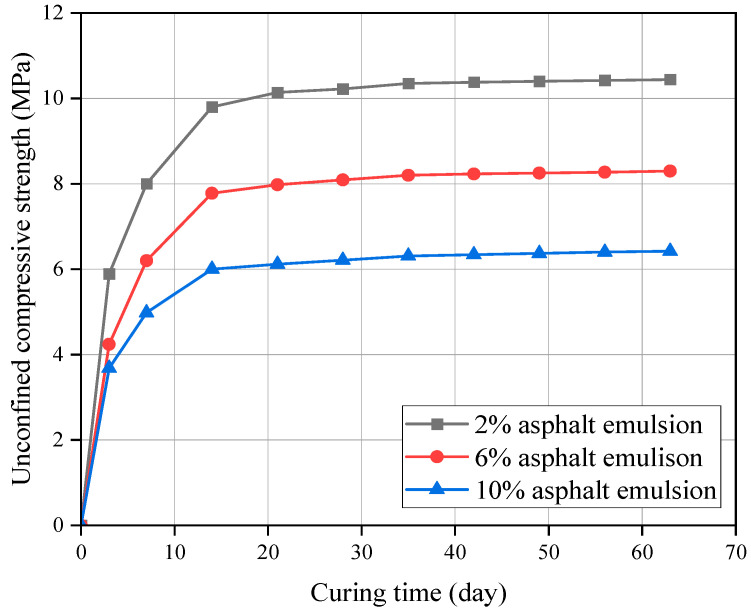
UCS of CSM-5 with 10% cement at different curing periods.

The UCS curves of CSM-5 mixtures with cement contents of 4% and 10% with different asphalt emulsion contents are shown in Figure 7 and Figure 8.

It can be seen from Figure 7 and Figure 8 that the UCS of CSM-5 mixtures with the cement contents of 4% and 10% increases gradually with the curing time under different asphalt emulsion contents. However, there are obvious differences in the growth rate of each growth stage, with faster growth in the early stage (3–28 d) and slower growth in the late stage (28–63 d). Due to the low strength of asphalt emulsion stabilized materials, the hydration of cement is the main reason for the formation of strength in cement-and-asphalt-emulsion-composite-stabilized-macadam. The content and hydration rate of four types of clinker minerals in cement were different (C3A and C3S react faster and, thus, play a certain role in promoting the formation of early strength; the reaction speed of C2S and C4AF is slow, which is the main guarantee for the strength growth in the later stage), which means that the cement-and-asphalt-emulsion-composite-stabilized-macadam show the characteristics of rapid strength growth in the early stage, and slow strength growth in the later stage.

### 4.2. Flexural Tensile Strength

#### 4.2.1. Influence of the Asphalt Emulsion Content

**Figure 9:**
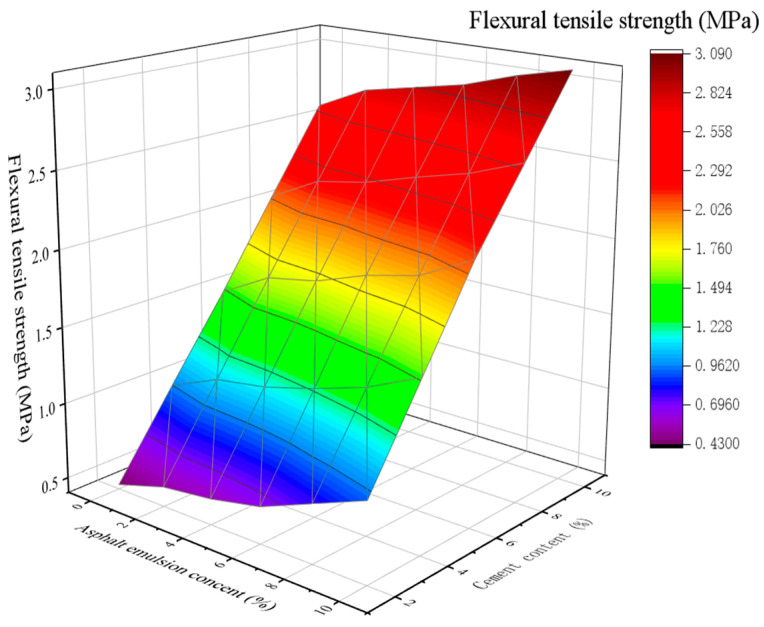
The 3D fitting of the asphalt emulsion content, cement content, and FTS of CSM-5.

**Figure 10:**
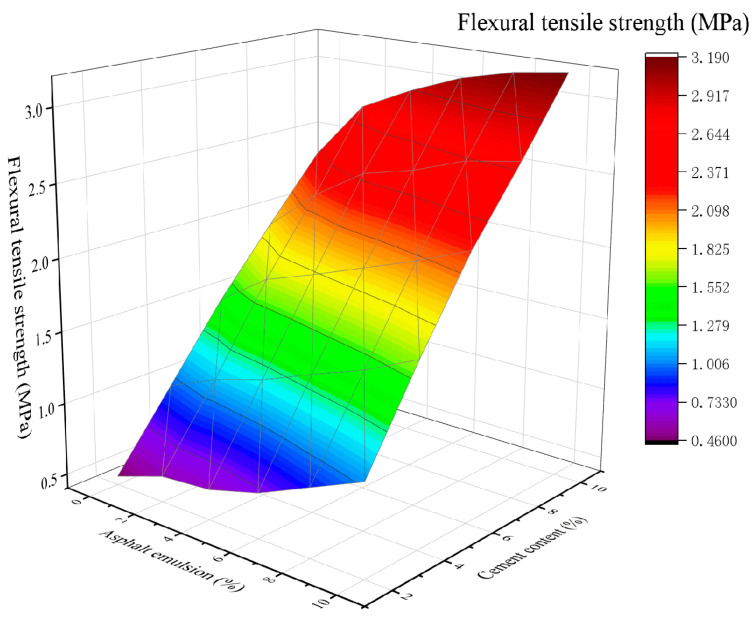
The 3D fitting of the asphalt emulsion content, cement content, and FTS of CSM-10.

The relationship between the FTS of CSM-5 and CSM-10 mixture and asphalt emulsion after curing for 28 d is shown in Figure 9 and Figure 10.

As seen in Figure 9 and in Figure 10, the higher the cement content, the greater the FTS of the CSM-5 and CSM-10 mixtures. At the same cement content, the FTS of the mixture gradually increased with the increase in asphalt emulsion content, and reached the maximum value at 10% of asphalt emulsion. Although asphalt emulsion affects the cement hydration reaction, hydration products have significantly improved the stiffness of the asphalt film, which may have a positive effect on the FTS.

#### 4.2.2. Influence of Curing Periods

**Figure 11:**
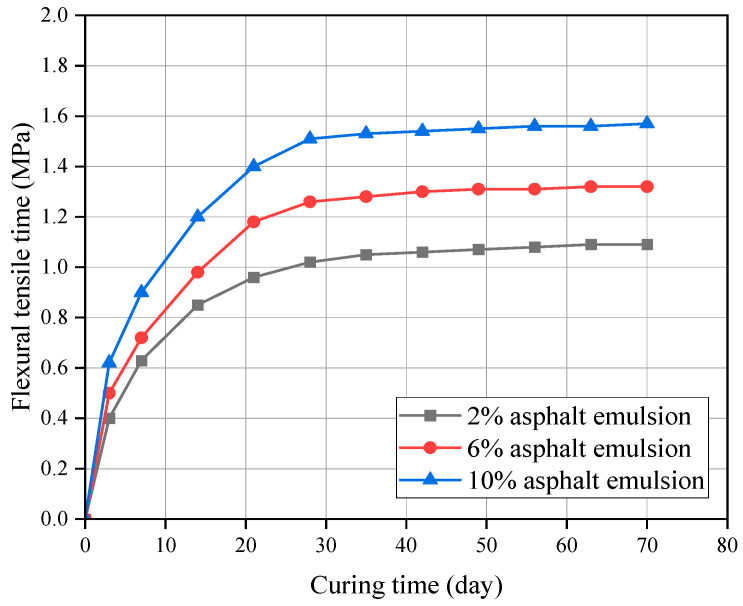
FTS of CSM-5 with 4% cement at different curing periods.

**Figure 12:**
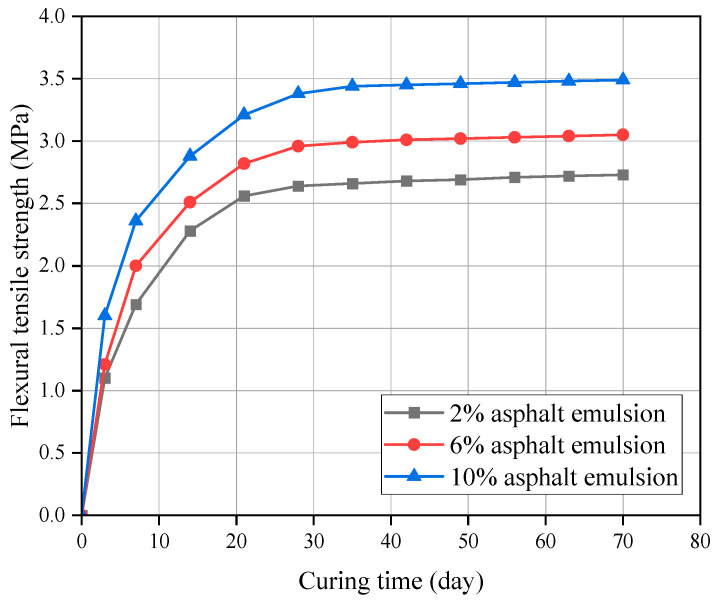
FTS of CSM-5 with 10% cement at different curing periods.

The FTS curves for CSM-5 mixes with 4% and 10% cement contents at different asphalt emulsion contents are shown in Figure 11 and Figure 12.

It can be seen from Figure 11 and Figure 12 that the FTS value of the CSM-5 mixture increased continuously with the extension of the curing time. However, there were obvious differences in the growth rate across the stages, among which the growth rate was faster in the early stage (3–28 d) and gradually slowed down in the later stage (28–70 d). These conclusions were similar to the analysis conclusions for UCS. The only difference was that FTS values were positively correlated with the asphalt emulsion content.

### 4.3. Structural Defect Coefficient UCS/FTS

**Figure 13:**
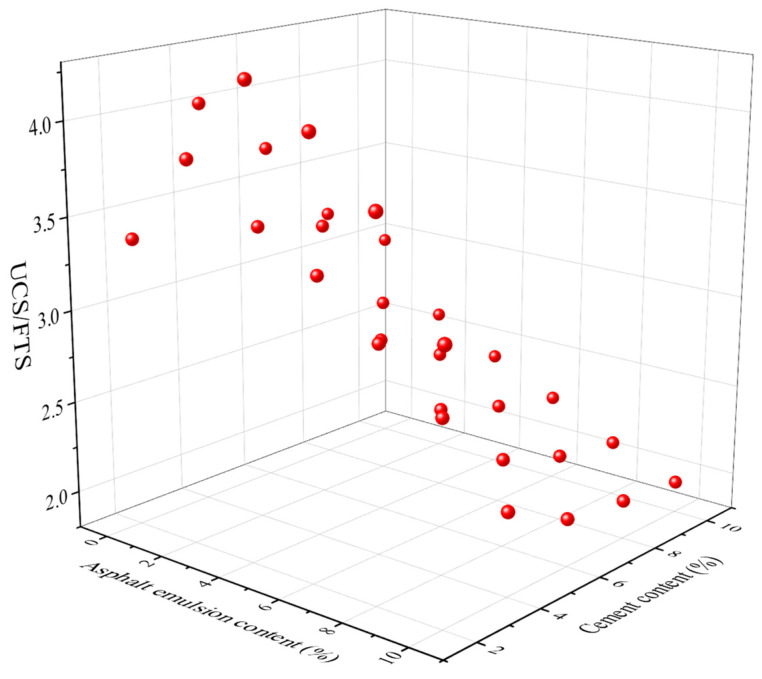
The relationship between the structural defect coefficient UCS/FTS of CSM-5 and the cement content and the asphalt emulsion content.

**Figure 14:**
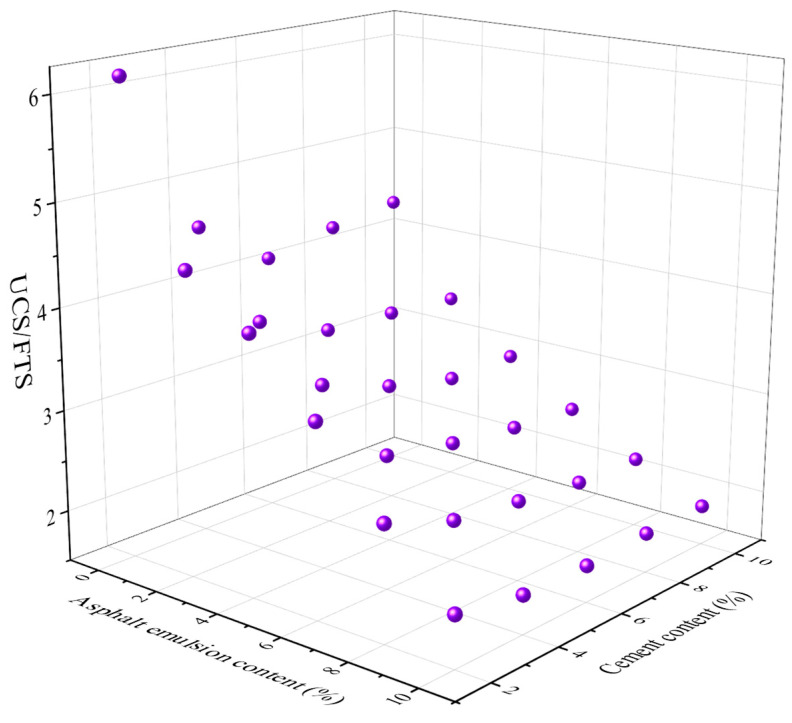
The relationship between the structural defect coefficient UCS/FTS of CSM-10 and the cement content and the asphalt emulsion content.

The structural defect coefficient can be used to evaluate the material condition quickly and effectively. The structural defect coefficient UCS/FTS for CSM-5 and CSM-10 with different cement contents and different asphalt emulsion contents are shown in Figure 13 and Figure 14.

It can be seen from Figure 13 and Figure 14 that with the increase in cement content, the structural defect coefficients of cm-5 and cm-10 decrease, which is because the cement content increases the cementitious properties between the aggregates and the hydration products of cement fill the voids between the coarse aggregates, thus decreasing the structural defect coefficients. The increase in the asphalt emulsion content also leads to a reduction in the structural defect coefficient. The addition of asphalt emulsion also plays a role in filling the voids. However, the main reason is that the addition of asphalt emulsion reduces the UCS of CSM and increases the FTS of CSM.

### 4.4. Elastic Modulus

**Figure 15:**
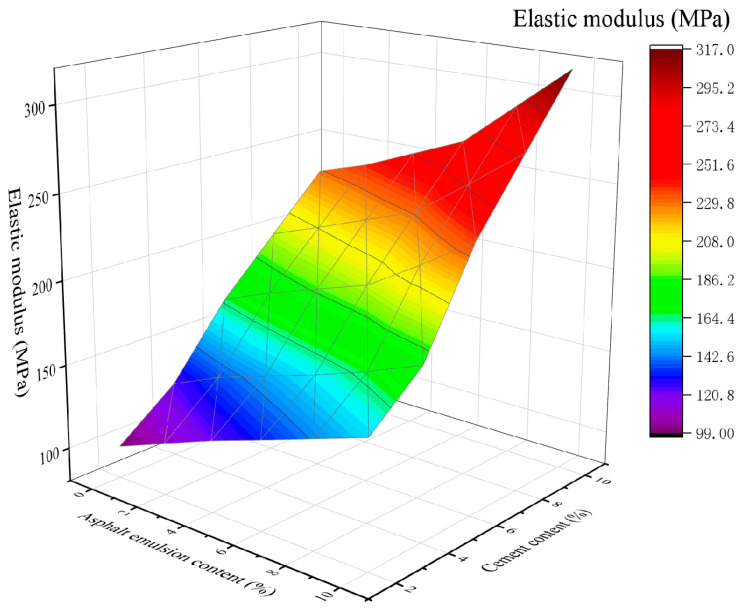
The 3D fitting of the asphalt emulsion content, cement content, and elastic modulus of CSM-5.

**Figure 16:**
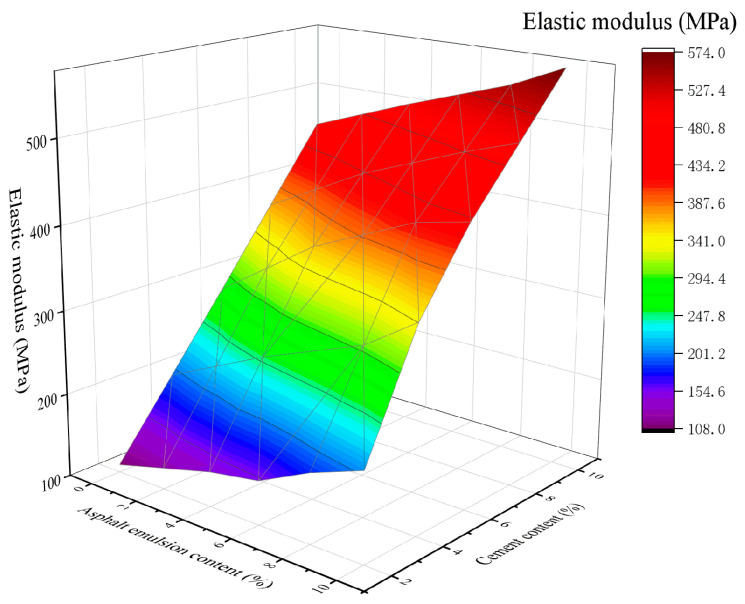
The 3D fitting of the asphalt emulsion content, cement content, and elastic modulus of CSM-10.

Figure 15 and Figure 16 show the variation of the elastic modulus in the mixtures in CSM-5 and CSM-10 as a function of the cement and asphalt emulsion content.

From Figure 15 and Figure 16, it can be seen that the elastic modulus of CSM-5 and CSM-10 mixtures are positively correlated with the cement content and asphalt emulsion content. As can be seen in Figure 15, for CSM-5 mixtures with cement content of 2%, 4%, 6%, 8%, and 10%, the elastic modulus increases as the asphalt emulsion content increases from 0% to 10%: 1.55, 1.45, 1.42, 1.40 and 1.38 times, respectively. As can be seen in Figure 16, for CSM-10 mixtures with cement contents of 2%, 4%, 6%, 8%. and 10%, the elastic modulus increases as the asphalt emulsion content increases from 0% to 10%: 1.88, 1.75, 1.58, 1.42, and 1.32 times, respectively.

### 4.5. Frost Resistance

**Figure 17:**
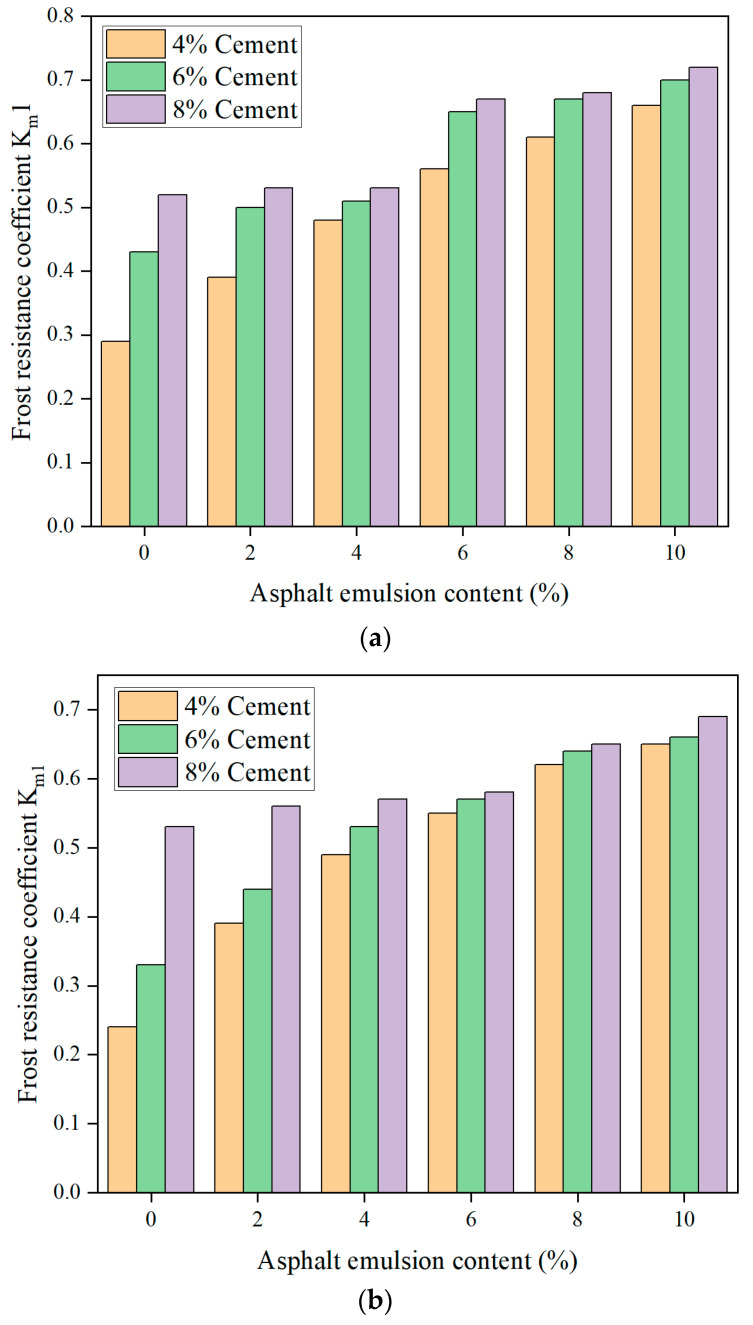
The relationship between the frost resistance coefficient K_m1_ and the asphalt emulsion content: (**a**) CSM-5, (**b**) CSM-10.

During the study of frost resistance, CSM-5 and CSM-10 mixtures stabilized with different cement contents (4%, 6%, and 8%) and asphalt emulsion contents (0%, 2%, 4%, 6%, 8%, and 10%) were subjected to 5, 10, 15, 20, and 30 freeze–thaw cycles, respectively. The frost resistance of CSM-5 and CSM-10 was evaluated using the frost resistance coefficient K_m1_ defined according to the splitting strength of the specimens before and after 30 freeze–thaw cycle tests, and the results are shown in Figure 17.

From Figure 17, it can be seen that the frost resistance coefficient K_m1_ of the mix increased with the increase in cement content under the same asphalt emulsion content. The frost resistance coefficient K_m1_ of CSM gradually increased with the increase in asphalt emulsion content under the same cement admixture, indicating that the asphalt emulsion admixture played a positive role in the frost resistance performance of CSM. This result was consistent with the test results for the FTS and elastic modulus of CSM.

## 5. Conclusions

The UCS, FTS, elastic modulus, and frost resistance of CSM were evaluated via the addition of asphalt emulsion into CSM, and the following conclusions were drawn:

1. With the increase in the asphalt emulsion content in CSM, the UCS is inversely proportional to the asphalt emulsion content. However, with the same asphalt emulsion content, the UCS of CSM gradually increases with the increase in cement content. With the increase in asphalt emulsion content in CSM, the FTS and elastic modulus also increase.
2. The UCS and FTS of CSM gradually increased with the curing time. In particular, the growth rate is the largest in the first 28 d, and the growth rate gradually slows down after 28 d.
3. With the increase in cement content and asphalt emulsion content, the structural defect coefficient UCS/FTS of CSM decreases gradually.
4. Since the addition of asphalt emulsion reduces the UCS of CSM, the frost resistance is evaluated according to the splitting strength of CSM before and after freezing and thawing. With the increase in the cement content and asphalt emulsion content, the frost resistance of CSM increases.

In future research, we will mainly study the shrinkage characteristics, maximum structural strength, and low-temperature cracking resistance of CSM with asphalt emulsion, so as to promote and apply it in high, cold areas.

## Figures and Tables

**Table 1:** Properties of Portland cement.

Properties	283-Day Compressive Strength (MPa)	3-Day Flexural Strength (MPa)	Initial Setting Time (min)	Final Setting Time (h)	Soundness
Measured value	28.2	6.1	180	210	qualified

**Table 2:** The physical properties of granite aggregates.

Test Items	Particle Size (mm)	
	0–5	5–10
Bulk density (kg/cm^3^)	1500	1417
Average density (g/cm^3^)	-	2.68
True density (g/cm^3^)	2.77	2.79
Porosity (%)	-	0.4
The content of lamellar and needle-shaped grains (%)	5	18
Clay content in lumps (%)	0	0
Crushing grade	650	3.1/1400
Wear grade	-	C_T_-1
Humidity (%)	1.2	0.15
Water absorption (%)	-	0.5

**Table 3:** The physical properties of the asphalt emulsion.

Properties	Specifications	Values
Residue via distillation (%)	50–70	55
PH	1.50–6.50	5.50
Uniformity (%)	0.5	0.15
Mineral viscosity per t% (c):		
- 20 °C.	65	45
- 25 °C.	65	40
Storage stability (%):		
- after 7 days.	0.80	0.30
- after 14 days.	1.20	0.40
Adhesion of the binder isolated from the bitumen emulsion with the surface of the crushed stone (%)	75	90

## Data Availability

Data are contained within the article.
